# Determining the most accurate 16S rRNA hypervariable region for taxonomic identification from respiratory samples

**DOI:** 10.1038/s41598-023-30764-z

**Published:** 2023-03-09

**Authors:** Ruben López-Aladid, Laia Fernández-Barat, Victoria Alcaraz-Serrano, Leticia Bueno-Freire, Nil Vázquez, Roque Pastor-Ibáñez, Andrea Palomeque, Patricia Oscanoa, Antoni Torres

**Affiliations:** 1 Cellex Laboratory, CibeRes (Centro de Investigación Biomédica en Red de Enfermedades Respiratorias, 06/06/0028), Institut d’Investigacions Biomèdiques August Pi I Sunyer (IDIBAPS), Barcelona, Spain; 2 School of Medicine, University of Barcelona, Barcelona, Spain; 3 Department of Pneumology, Thorax Institute, Hospital Clinic of Barcelona, Barcelona, Spain; 4 Group of Genomics and Pharmacogenomics in HIV, Laboratory of Retrovirology and Viral Immunopathogenesis, Hospital Clinic of Barcelona, Barcelona, Spain

**Keywords:** Diseases, Microbiology, Microbial communities, Microbiome

## Abstract

16S rRNA gene profiling, which contains nine hypervariable regions (V1–V9), is the gold standard for identifying taxonomic units by high-throughput sequencing. Microbiome studies combine two or more region sequences (usually V3–V4) to increase the resolving power for identifying bacterial taxa. We compare the resolving powers of V1–V2, V3–V4, V5–V7, and V7–V9 to improve microbiome analyses in sputum samples from patients with chronic respiratory diseases. DNA were isolated from 33 human sputum samples, and libraries were created using a QIASeq screening panel intended for Illumina platforms (16S/ITS; Qiagen Hilden, Germany). The analysis included a mock community as a microbial standard control (ZymoBIOMICS). We used the Deblur algorithm to identify bacterial amplicon sequence variants (ASVs) at the genus level. Alpha diversity was significantly higher for V1–V2, V3–V4, and V5–V7 compared with V7–V9, and significant compositional dissimilarities in the V1–V2 and V7–V9 analyses versus the V3–V4 and V5–V7 analyses. A cladogram confirmed these compositional differences, with the latter two being very similar in composition. The combined hypervariable regions showed significant differences when discriminating between the relative abundances of bacterial genera. The area under the curve revealed that V1–V2 had the highest resolving power for accurately identifying respiratory bacterial taxa from sputum samples. Our study confirms that 16S rRNA hypervariable regions provide significant differences for taxonomic identification in sputum. Comparing the taxa of microbial community standard control with the taxa samples, V1–V2 combination exhibits the most sensitivity and specificity. Thus, while third generation full-length 16S rRNA sequencing platforms become more available, the V1–V2 hypervariable regions can be used for taxonomic identification in sputum.

## Introduction

The airway microbiota plays a crucial role in the development, progression, and exacerbation of chronic respiratory diseases like non-cystic fibrosis bronchiectasis. Indeed, dysbiosis alters the lung structure and affects the pulmonary immune response, diseases course, and therapeutic efficacy^1,2^. However, the composition of the microbiota and the biodiversity gradient decrease in the lower airways^1^, where the environment is nutrient-poor and constant microbiome turnover results from natural barriers such as coughing, mucociliary clearance, and host defense mechanisms^3,4^. The main phyla in healthy lungs are Firmicutes, Bacteroidetes, and Proteobacteria, with the genera *Streptococcus*, *Prevotella*, and *Veillonella* predominating and the genera *Haemophilus* and *Neisseria* less abundant^1,5,6^.

High-throughput 16S rRNA gene sequencing now has proven utility in the field of microbiology, becoming the most common method in the study of bacterial phylogeny and taxonomy^7^. 16S profiling can be used to survey most bacteria because it is universally present in the genomes of all prokaryotes. Moreover, genomic identification could replace conventional pathogen identification by morphology, staining, and metabolic criteria to improve diagnosis and better predict clinical outcomes^8^. High-throughput sequencing, such as the whole genome shotgun method, can also shed light on the actual functions of bacteria, together with their drug sensitivity and resistance, relationships with other pathogens, and roles in homeostasis and pathology. High-throughput sequencing by 16S rRNA gene profiling overcomes the limitations of standard of care bacterial cultures, offering a realistic picture of not only the dominating bacteria but also the other non-culturable bacteria. This ecological perspective and its clinical implications have become a focus of research attention^9^.

Everyone has a unique ecological balance of microorganisms. This microbiota profile has co-evolved in the host to colonize its mucosal tissues by establishing a symbiotic relationship. The term microbiome is used to refer to the entire habitat, including their microorganisms, genomes, and the surrounding environment^10,11^. However, technical variations among 16S profiling studies, such as the DNA extraction protocols, primers, PCR conditions, hypervariable regions, taxonomic resolution, and sequencing platforms can significantly affect the taxonomic classification^12,13^.

Depending on the hypervariable regions used for 16S rRNA profiling, different taxonomic resolutions and 16S rRNA gene frequencies are obtained across each ecological niche. Although one region may be suitable for a given body site, it might not be as accurate elsewhere. For example, the V1 region (nucleotide position: 69–99) can be used to identify pathogenic *Streptococcus sp*. and to differentiate between *Staphylococcus aureus* and coagulase-negative *Staphylococcus*^14,15^. We also known that the V4, V5, and V6 regions have the highest functionality in the ribosome and are highly conserved (especially the V4 region)^12^. By contrast, the V2 and V8 regions are structural and show little functionality in the ribosome^13^, like the V3 and V7 regions, which also have no role or functionality.

Combined two or more hypervariable regions has been suggested to increase their resolving power in the identification of bacteria at the genus level^16^. However, for the evaluation of respiratory samples, no standardized methods exist for the use of several hypervariable regions to compare sensitivity limits for diversity and richness in control mock communities and standard of care cultures. This is needed to ensure the reliability and reproducibility of the results. The actual clinical advantages of 16S profiling lie not in microbial diagnostics but in the exploration of non-culturable organisms. This can uncover precise taxonomic levels and how they interact to help decipher their associations with clinical outcomes and host immune phenotypes.

**Figure 1:**
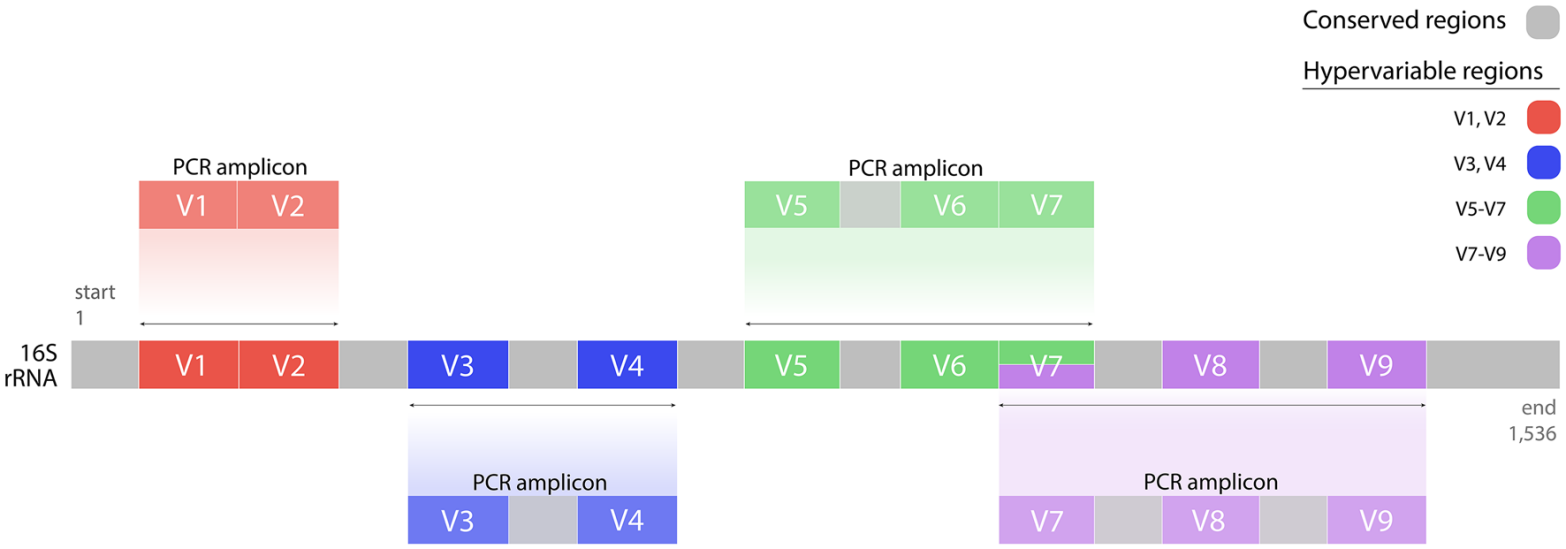
Hypervariable region strategy on 16S rRNA. A summary of the four hypervariable region strategies for amplification of variable regions V1–V2, V3–V4, V5–V7, and V7–V9 in the 16S rRNA gene (positioning is based on the *E.coli* 16S rRNA gene).

The aim of this study was to compare combinations of hypervariable regions (i.e., V1–V2, V3–V4, V5–V7 and V7–V9) to optimize sputum microbiome analysis in patients with underlying chronic respiratory disease (Fig. 1).

## Results

This study is based on amplicon sequence variants (ASVs), enabling slightly stronger detection of bacterial diversity because operational taxonomic units (OTUs) do not provide accurate measurements of sequence variation.

**Figure 2:**
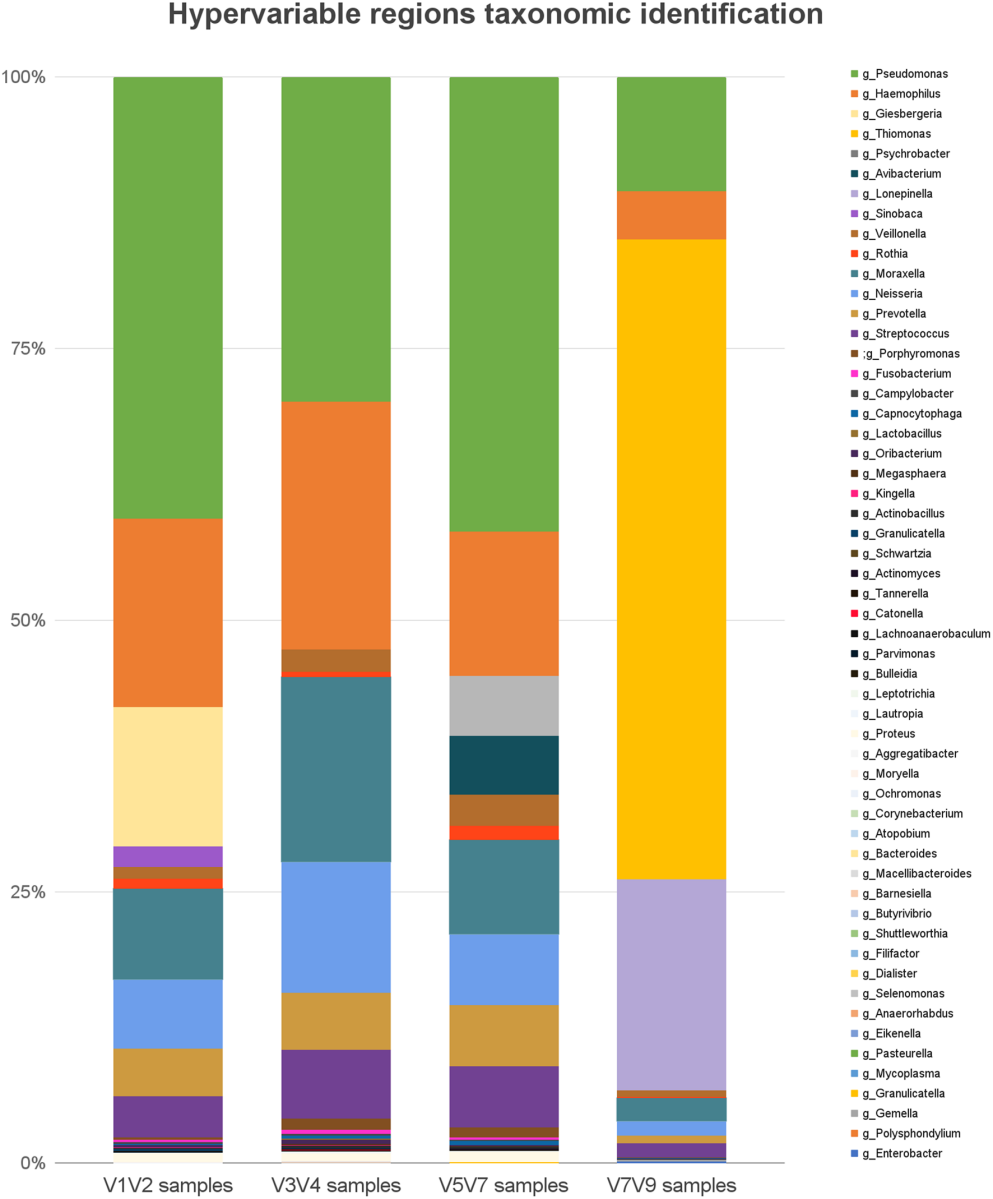
Amplicon sequence variants according to the different hypervariable regions of the 16S rRNA gene. Samples are grouped and averaged by hypervariable region, with taxonomic composition shown at the genus level. Each column represents a hypervariable region and each color represents the percentage of the total sample contributed by each taxonomy.

FastQC was used to obtain quality sequences with Q30 and to observe the number of reads per sample, bases per sequence, sequence distribution length, and scores per sequence. During quality control and read preprocessing from 33 samples, we obtained an average of 100,000 sequences per sample. This study obtained 114 taxonomies with 1,568,227 ASVs per region, which in turn, contained different taxa at the genus level. However, the total number of ASVs was the same for all hypervariable regions. Consistent without our findings in the ZymoBIOMICS Microbial Community control (Supplementary Material S1), each hypervariable region produced different relative frequencies for the genus *Pseudomonas* from major to minor (V1–V2, V3–V4, V5–V7, and V7–V9; Fig. 2).

**Figure 3:**
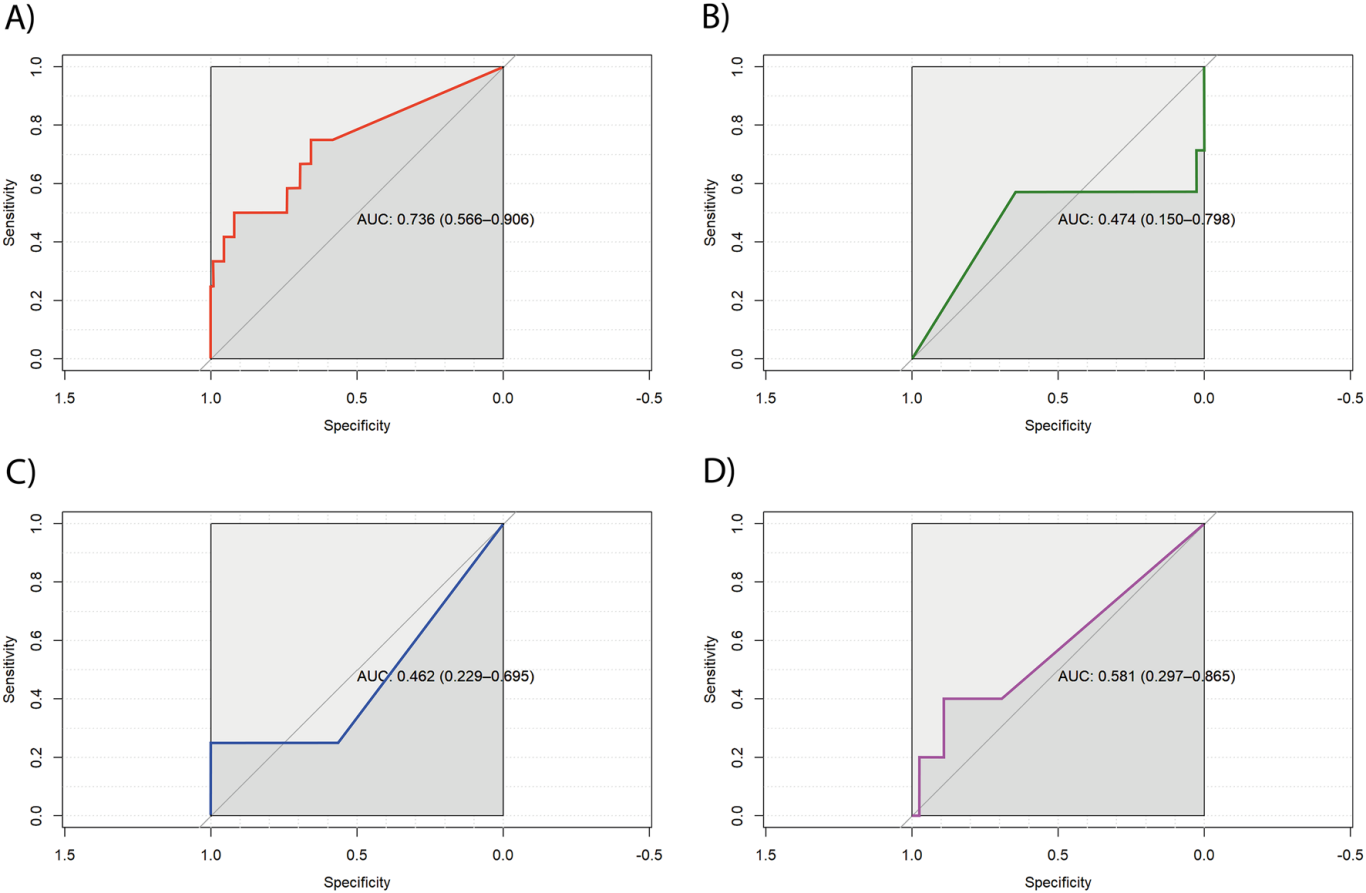
ROC curves for the hypervariable region validated with a mock community microbial standard control. The cross-validation accuracy of the microbiota classifier is depicted by the ROC curve for the bacterial genera obtained: (**A**) AUC for V1–V2 = 0.736 (0.566–0.906); (**B**) AUC for V3–V4 = 0.474 (0.150–0.798); (**C**) AUC for V5–V7 = 0.462 (0.229–0.695); and (**D**) AUC for V7–V9 = 0.581 (0.297–0.865). As shown, a strong association existed between a specific microbiome genus composition and the V1–V2 hypervariable region. *AUC* area under the curve, *ROC*, receiver operating characteristic.

We used a receiver operating characteristic (ROC) curve for cross-validation accuracy of the microbiota classifier in the microbial standard control (ZymoBIOMICS). Then, we performed pair-wise comparisons with each combined hypervariable region (V1–V2, V3–V4, V5–V7, and V7–V9) to compare sensitivity and specificity between the regions and the specific microbial standard control ROC curve using as a microbiota classifier Geengenes database for cross-validation between ZymoBIOMICS and samples. The V1–V2 combined region had a significant area under the curve (AUC) of 0.736 with an interquartile range (IQR) of 0.566–0.906 (Fig. 3A) while V3–V4, V5–V7 and V7–V9 (Fig. 3B,C and D respectively), hadn’t a significant AUC. These results suggest that the V1–V2 hypervariable region exhibits the highest sensitivity and specificity for respiratory microbiota.

### Alpha diversity among the combined hypervariable regions of 16S rRNA

**Figure 4:**
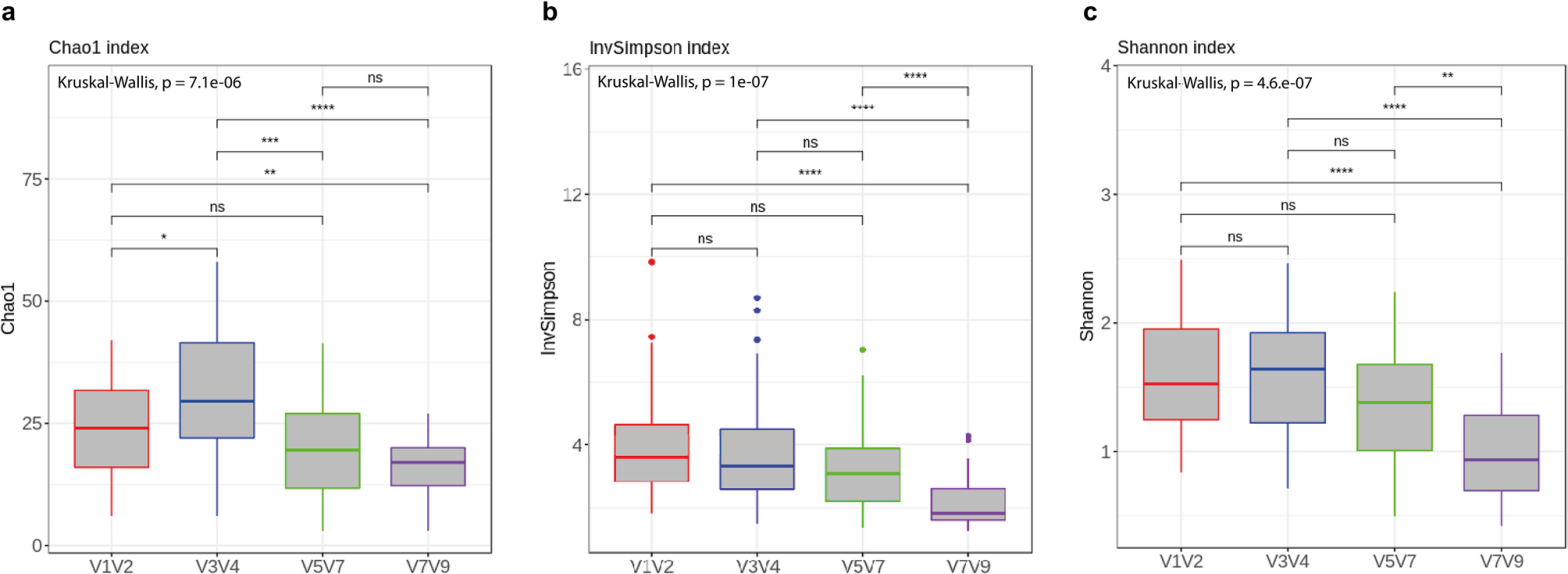
Alpha diversity in sputum samples compared with different combined hypervariable regions in 16S rRNA. Alpha diversity, measured by the Chao1 (Fig. 3A), inverse Simpson (Fig. 3B), and Shannon(Fig. 3C) diversity indices, is plotted for the hypervariable regions V1–V2 (red), V3–V4 (blue), V5–V7 (green), and V7–V9 (purple). The line inside the boxplot represents the median, with the lowest and highest values within the 1.5 interquartile range represented by the whiskers. All diversity indices were significantly decreased in V7–V9: pShannon = 4.6 E-07, pinvSimpson = 1 E-07, and pChao1 = 7.1 E-06.

The Shannon and inverse Simpson indices were significantly higher for V1–V2, V3–V4, and V5–V7 compared with V7–V9. Figure 4 shows the alpha diversity measurements for the different hypervariable regions to observe significant differences between groups based on the Kruskal–Wallis test. Alpha diversity was lower in the V7–V9 group compared with the other regions. The Simpson index is considered more of a dominance index because it accounts for the proportion of each species in a sample. As shown in Fig. 4, the V7–V9 combined region had a significantly lower proportion of species (*p* < 0.0001) than the other regions. By contrast, the Shannon index indicates the randomness at a site and considers both species richness and equitability in the sample distribution. This index revealed that V7–V9 had the lowest entropy (*p* < 0.0001). Finally, Chao1 indexes demonstrate the richness of bacterial communities. Figure 4 shows the highest Chao1 was found in V3–V4, whereas V7–V9 had a significantly lower Chao1 (*p* < 0.0001) .

### Beta diversity among the combined hypervariable regions of 16S rRNA

**Figure 5:**
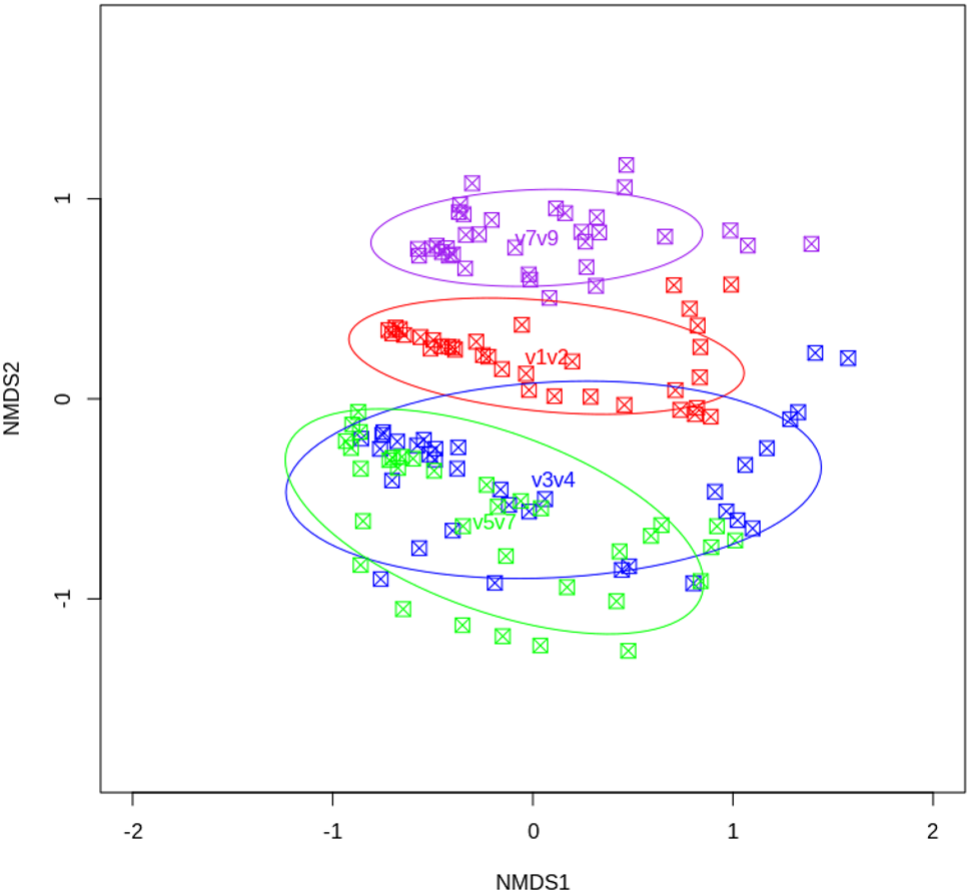
NMDS ordination of the Bray–Curtis dissimilarity index for different combined hypervariable regions in 16S rRNA. The groups identified by different combined regions present compositional differences. Each sample is represented by a dot: green for V5–V7; blue for V3–V4; red for V1–V2; and purple for V7–V9. Sample proximity indicates similarity (closer = more similar). *NMDS* Non-metric multidimensional scaling.

Figure 5 shows the results for non-metric multidimensional scaling (NMDS) ordination of Bray–Curtis dissimilarities between different sites (R2 = 0.44, pAdonis < 0.001), where NMDS axis “0” and “1” indicate the minimum and maximum dissimilarities between sites, respectively. Our findings show that the hypervariable regions have 44% compositional dissimilarities. In particular, the V3–V4 and V5–V7 regions overlapped in both NMDS1 (X axis) and NMDS2 (Y axis) dimensions, indicating compositional similarity. However, V7–V9 (purple dots) and V1–V2 (red dots) show large dissimilarities, as do V3–V4 (blue dots) and V5–V7 (green dots), indicating compositional differences in NMDS2. The residual stress is plotted in supplementary material S2.

### Linear discriminant analysis effect size score for taxonomic abundance

**Figure 6:**
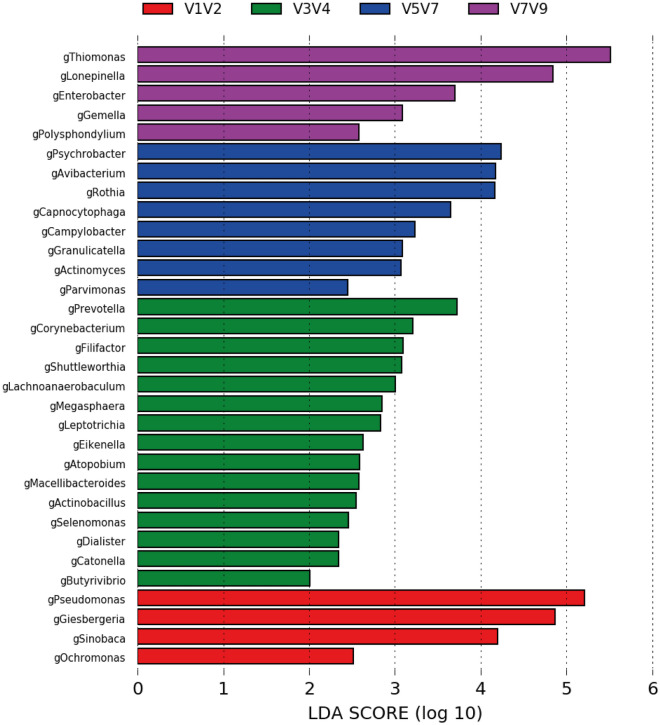
LEfSe for differentially abundant combined regions among sputum samples. LDA finds taxa that are significantly more abundant per group. Bars represent the most abundant bacterial taxa at the genus level in sputum samples from patients with bronchiectasis: V1–V2 (red), V3–V4 (green), V5–V7 (blue), and V7–V9 (purple). The bar size (X axis) from LEfSe represents the effect size of the differential abundance of taxa for the each region (statistical significance if > 2 log). *LDA* linear discriminant analysis, *LEfSe* linear discriminant analysis effect size.

**Figure 7:**
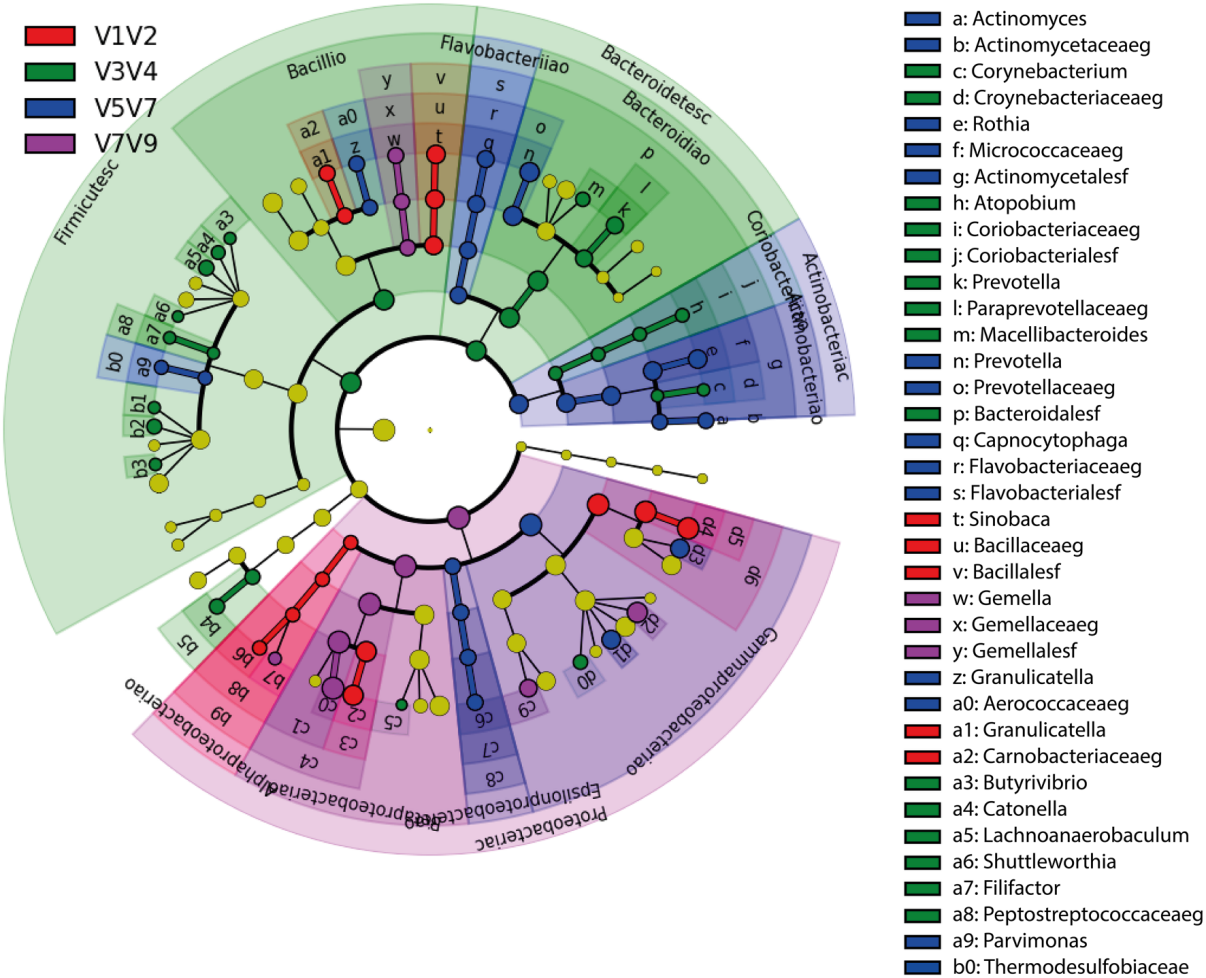
Cladogram of the LEfSe results for the different combined regions in 16S rRNA. The cladogram shows the microbial species with significant differences in the LDA score between the analyzed groups. Colors indicate the taxa identified by the different combined regions: V1–V2 (red), V3–V4 (green), V5–V7 (blue), and V7–V9 (purple). Species classification at the class, order, family, and genus levels is shown from the inside to the outside. Yellow nodes represent species with no significant difference, indicating the cladograms that overlapped between hypervariable regions.. At the end of each taxa names, the taxonomic level appears in the LEFSE output, where “o” represents the order taxonomic level, “c” the class level, “f” the family level, “p” the phylum level and “s” the of kind. *LDA* linear discriminant analysis, *LEfSe* linear discriminant analysis effect size.

Linear discriminant analysis (LDA) effect size (LEfSe) is currently used as a biomarker discovery tool for high-dimensional data. In this study, taxonomic abundance at the genus level explained differences between hypervariable regions by coupling standard tests for statistical significance with additional tests for encoding biological consistency and effect relevance. We found that LEfSe revealed differences in the discriminative power of each hypervariable region (Figs. 6 and 7): V1–V2 was discriminant for the relative abundances of *Pseudomonas, Glesbergeria, Sinobaca, and Ochromonas*; V3–V4, for *Prevotella, Corynebacterium, Filifactor, Shuttleworthia, Lachnoanaerobium, Megasphaera, Leptotrichia, Eikenella, Atopobium*, *Mecelli Bacteroides, Actinobacillus, Selomonas, Dialister, and Catonella*; V5–V7, for *Psycrobacter, Avibacterium, Othia, Capnocytophaga, Campylobacter, Granulicatella, Actinomyces, and Parvimonas*; and V7–V9, for *Thiomonas, Lonepinella, Gemella, Enterobacter* and *Polysphondilium*.

## Discussion

The main finding of this study is that each 16S rRNA hypervariable region provides significant differences in taxonomic identification. The V1–V2 region had the highest resolving power for accurately identifying respiratory bacterial taxa in sputum samples. The highest concordance between samples and the ZymoBIOMICS microbial community control was also found in the V1–V2 region, followed by V3–V4, V5–V7, and V7–V9, for the relative frequencies of *Pseudomonas,* the dominant genus in our samples. V1–V2 also produced the best ROC curve (AUC, 0.736; IQR, 0.566–0.906), confirming that it had higher sensitivity and specificity for sputum microbiome analyses. Combined regions V1–V2, V3–V4, and V5–V7 presented similar alpha diversities in contrast with V7–V9, and V1–V2 and V3–V4 presented the lowest dissimilarity in beta diversity. Finally, the fact that by LEfSe the only region that obtained Pseudomonas genus was V1–V2 confirmed our results with Zymobiomics given that Pseudomonas was confirmed previously by bacterial culture in all patients.

To date, no studies have reported the resolving power of 16S rRNA hypervariable regions for respiratory microbiome analyses. In the study of vaginal microbiota by Sirichoat et al. (2021), V3 gave the greatest richness and diversity, followed by V6–V7 and V4, while V9 gave the lowest richness and diversity. Despite the use of single regions to achieve high coverage, it not only lowers the discriminatory power given the short length of the base pairs (bp)^17^ but also enhances the cumulative error rate^9^. We also found that V3–V4 and V5–V7 (comprising V6) presented the higher alpha index values^17^.

Garcia-Lopez et al. (2020) studied 24 hepatopancreatic and intestinal samples using the Greengenes and SILVA reference databases for clustering and taxonomic classification. They found that V3–V4 resulted in the highest richness in alpha diversity, consistent with our findings. Together with our results, these findings suggest that taxa identification requires approximately 500 bp to achieve balance between length and depth of coverage, combining one of the most conserved regions (V4) with one of the most variable regions (V3)^18^.

Yang et al. (2016) evaluated the sensitivity of different 16S rRNA hypervariable regions as biomarkers of different bacterial phyla, using the geodesic distance and the consensus agglomerative hierarchical clustering methods. The V4–V6 combination represented the optimal hypervariable region for the phylogenetic study of new bacterial phyla^19^. Nevertheless, they used a different methodology to ours, necessitating further standardized methodology studies to compare the utility of the V4–V6 and V1–V2 regions for confirming taxonomic identification.

The selection of combined hypervariable regions also depends on the use of published or in-house protocols^18–21^. Sperling et al. (2017) used the commercially available Ion 16S rRNA Gene High-throughput Sequencing (16S profiling) Kit, based on six amplicons representing 16S regions (V2, V3, V4, V6, V7, V8, and V9). They showed that the diversity patterns obtained by 16S microbiome surveys depended both on the amplicon and the protocol used^22^. In addition, microbial composition data differs with the DNA isolation method, library preparation, and sequencing platform^23^. Commercial kits exist for 16S profiling analysis that usually contain PCR primers for the V3 and V4 regions and shotgun protocols^24^.

Our study showed four combinations of the 16S rRNA gene hypervariable regions (V1–V2, V3–V4, V5–V7, V7–V9) that can be assessed with commercially available kits. These can detect bacteria in samples with low microbial density or with contamination by host DNA, such as human tissue and low biomass samples that are particularly susceptible to bias. In addition, the screening panel enabled comparison of the sensitivity of each combined hypervariable region in relation to diversity, richness, and relative abundances in sputum samples. Most of the other studies discussed here lack direct comparability because richness or entropy vary depending on the actual composition, which is niche dependent. Therefore, this study is the first to evaluate the taxonomic resolution of different hypervariable regions in sputum. Further studies of sputum samples are needed to confirm our findings.

This study has several limitations. First, DADA2 identified more real variants and output sequences than other methods, despite Deblur being recommended for shorter bp fragments. Although we trimmed at 250 bp for this reason, the use of phase primers means that Deblur was an alternative option for analyzing multiple sequencing runs. Second, despite the availability of more updated versions of SILVA, the different regions were trained with Greengenes. Another limitation of this study was that mock microbial communities there are only 10 species and this fact affects in the sensitivity and specificity related with taxonomic classification. However, this should not affect the comparisons between different hypervariable regions because the bias in taxonomic identification might affect each region equally. Third, the 300 bp length limit of the Illumna MiSeq platform limits 16S rRNA profiling, emphasizing the importance of identifying the best hypervariable region for taxonomic identification.

## Conclusions

Our study confirms that each 16S rRNA hypervariable region provides significant differences in taxonomic identification. Hypervariable combined region V1–V2 appears to have the highest sensitivity and specificity, exhibiting the highest resolving power for taxonomy identification in sputum. Although further research is encouraged to elucidate the resolving power of each hypervariable region compared to full-length 16S rRNA sequencing, suitable Nanopore sequencing platforms need to become more widely available for clinical application.

## Methods

This prospective observational study (NCT04803695) complied with the Declaration of Helsinki (current version, Fortaleza, Brazil, October 2013). Our institution’s Internal Review Board approved the study, and all patients gave their written informed consent (No. HCB/2018/0236, Hospital Clinic Barcelona).

### Sputum samples and DNA extraction

Thirty-three sputum samples were collected from patients with bronchiectasis from July 2017 to October 2019. Sputum was frozen at –80ºC immediately after collection and DNA extraction was performed using the Sputum DNA Isolation Kit, according to the manufacturer’s instructions (Norgen Corp., Canada). Subsequently, DNA was quantified using Qubit (Thermo Fisher Scientific, MA, USA).

Ethics approval and consent to participate was granted by the Hospital Clinic of Barcelona (HCB/2018/0236). All patients signed an informed consent in the Hospital Clinic of Barcelona.

### PCR amplification and sequencing

The preparation of the DNA libraries was based on hypervariable regions V1–V2, V3–V4, V5–V7 and V7–V9 of the 16S rRNA gene using the QIAseq 16S/ITS Screening Panel (QIAGen Hilden, Germany), as summarized in Fig. 1. The DNA libraries were created and purified, removing short fragments (non-target products) with AMPure XP Beads (Agencourt Bioscience, Waltham, MA, USA). The libraries were sequenced with the MiSeq Sequencing Platform, using a V3 sequencing kit for 2 × 300 bp paired end reads (Illumina, San Diego, CA, USA).

A ZymoBIOMICS microbial community standard control was used as a positive control. ZymoBIOMICS provides information on the most representative species available online (https://www.zymoresearch.com/collections/ZymoBIOMICS-microbial-community-standards/products/ZymoBIOMICS-microbial-community-standard) (See Supplemental material S1). In accordance with QIAGEN’s ISO-certified Quality Management System, each QIAseq 16S/ITS Panel, QIAseq 16S/ITS Index Kit, and QIAseq 16S/ITS Smart Control Kit had also been tested against predetermined specifications to ensure consistent product quality.

Purification of DNA was done through AMpure beads provided by Beckman Coulter (Benckman, USA). Purified DNA was used as a template for the library preparation, with primers targeting each combined hypervariable region of the 16S rRNA gene using a QIASeq Screening Panel, according to the manufacturer’s instructions (Qiagen, Hilden, Germany). The following amplification program was used: denaturation at 95 °C for 5 min; 20 cycles of denaturation at 95 °C for 30 s; primer annealing at 60 °C for 30 s, extended to 72 °C for 30 s; and final elongation at 72 °C for 5 min. A secondary amplification was performed to attach the QIAGEN index barcode.

Finally, the library preparation products were assessed on a 2100 Bioanalyzer system (Agilent, Palo Alto, CA, USA), and a DNA 7500 kit was used to determine library quality and size. The pool of DNA libraries was introduced into the MiSeq Sequencing Platform (Illumina, San Diego, CA, USA) for DNA sequencing, which was performed at the IDIBAPS Core Facility. The files obtained were in the *fastq* format for bioinformatics analyses.

### Microbiome analyses

Microbiome analysis involved the following steps. FastQC Version 0.11.9 assessed quality and the filtering of raw reads with a cut-off score of up to 30^11^. Sequence denoising was performed with the Deblur pipeline, which is intended for paired-end data, and the non-redundant reads were deleted. Deblur is an alternative method to OTU binning, and it generates more precise ASVs and ensures greater reliability of taxonomic identification^26^. The non-chimeric 16S rRNA database from QIIME 2 was used to identify chimeras in the reads, with a best hit similarity rate of less than 99%. Taxonomic assignments were obtained using the Greengenes database and collapsed at the genus level, as implemented in the QIIME 2 software.

Sequence data were clustered using the R packages *phyloseq* and *vegan* into groups based on different regions, before producing cladograms with the different groups. The alpha diversity indices and rarefaction curves were estimated in R (version 3.4.1) with compatible *phyloseq* and *vegan* packages. Alpha diversity was quantified with Chao1, the Shannon index, and the inverse Simpson index, which are associated with ASV richness, evenness, entropy, and dominance, respectively. Statistical significance was calculated by Kruskal–Wallis tests.

A stress plot was calculated to analyze beta diversity (see supplemental Material S3)., with the ecological distance metrics calculated with the Bray–Curtis dissimilarities index implemented in R packages (*vegan*, *metaMDS*, and *ggplot2* open access packages; see *Availability of Data and Materials*). An NMDS approach was used for graphics related to beta diversity (Fig. 5), based on a confidence interval of 0.95. In addition, ROC curve analysis was used to assess the sensitivity and specificity of each hypervariable region, using ZymoBIOMICS as a positive control, again with a confidence interval of 0.95.

LDA is used for effect size estimation because our experiments determined that it more accurately estimated biological consistency compared with the use of differences in group means/medians^30^.The LEfSe method was used to uncover the significantly different bacterial taxa among samples from three regions, with taxa showing significantly different abundances (LDA threshold > 2) among the four groups represented by colored dots (for phylum, class, order, family, and genus, from inside to outside). The LDA score for each biomarker was obtained by computing the logarithm (base 10) of this value after being scaled (1,10exp9). Regardless of the absolute values of the LDA score, this was used to rank biomarker relevance. LDA was additionally supported by bootstrapping (default 30-fold) with averaging to improve robustness^30^.

### Ethics approval and consent to participate

This prospective observational study (NCT04803695) complied with the Declaration of Helsinki (current version, Fortaleza, Brazil, October 2013) and the requirements of the 2007 Spanish Biomedical Research Act. Our institution’s Internal Review Board approved the study, and all patients gave their written informed consent (No. HCB/2018/0236, Hospital Clinic Barcelona).

## Supplementary Information

Supplementary Information 1.

Supplementary Information 2.

Supplementary Information 3.

## Data Availability

All data generated or analyzed during this study are available from a Github repository titled: “Hypervariable-regions-of-16S-rRNA-with-the-highest-resolving-power-for-taxonomic-identification”, from the following Github URL: (https://github.com/Ruben-MetagenomiXs/-Hypervariable-regions-of-16S-rRNA-with-the-highest-resolving-power-for-taxonomic-identification). Datasets analyzed during the current study are available in the repository Sequence Read Archive (SRA) titled: “Hypervariable Regions of 16S rRNA in sputum” in the following Bioproject of NCBI: [https://www.ncbi.nlm.nih.gov/bioproject/PRJNA915241]. Accession: PRJNA915241 ID: 915,241.
