# Supplementary material for: Determining the most accurate 16S rRNA hypervariable region for taxonomic identification from respiratory samples
Source: Sci Rep. 2023 Mar 9;13:3974. doi: 10.1038/s41598-023-30764-z (PMC9998635; doi:10.1038/s41598-023-30764-z)
Supplement: Supplementary file 1 — Supplementary Information 1. [file 41598_2023_30764_MOESM1_ESM.pdf]

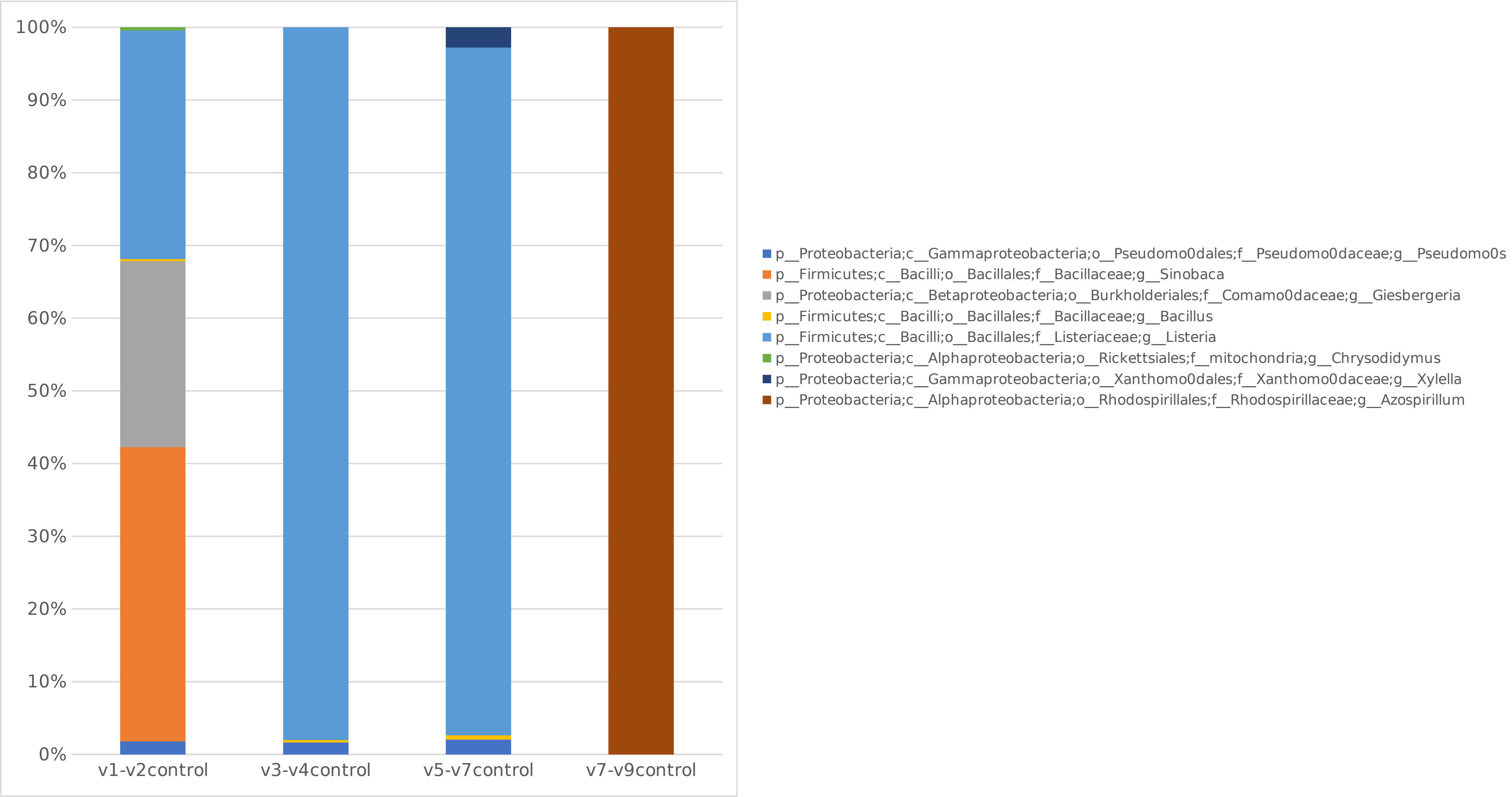
100%
90%
80%
70%
60%
50%
40%
30%
20%
10%
0%
v1-v2control
v3-v4control
v5-v7control
v7-v9control
p__Proteobacteria;c__Gammaproteobacteria;o__Pseudomo0dales;f__Pseudomo0daceae;g__Pseudomo0s
p__Firmicutes;c__Bacilli;o__Bacillales;f__Bacillaceae;g__Sinobaca
p__Proteobacteria;c__Betaproteobacteria;o__Burkholderiales;f__Comamo0daceae;g__Giesbergeria
p__Firmicutes;c__Bacilli;o__Bacillales;f__Bacillaceae;g__Bacillus
p__Firmicutes;c__Bacilli;o__Bacillales;f__Listeriaceae;g__Listeria
p__Proteobacteria;c__Alphaproteobacteria;o__Rickettsiales;f__mitochondria;g__Chrysodidymus
p__Proteobacteria;c__Gammaproteobacteria;o__Xanthomo0dales;f__Xanthomo0daceae;g__Xylella
p__Proteobacteria;c__Alphaproteobacteria;o__Rhodospirillales;f__Rhodospirillaceae;g__Azospirillum
