# Supplementary figures and images for: Determining the most accurate 16S rRNA hypervariable region for taxonomic identification from respiratory samples
Source: Sci Rep. 2023 Mar 9;13:3974. doi: 10.1038/s41598-023-30764-z (PMC9998635; doi:10.1038/s41598-023-30764-z)

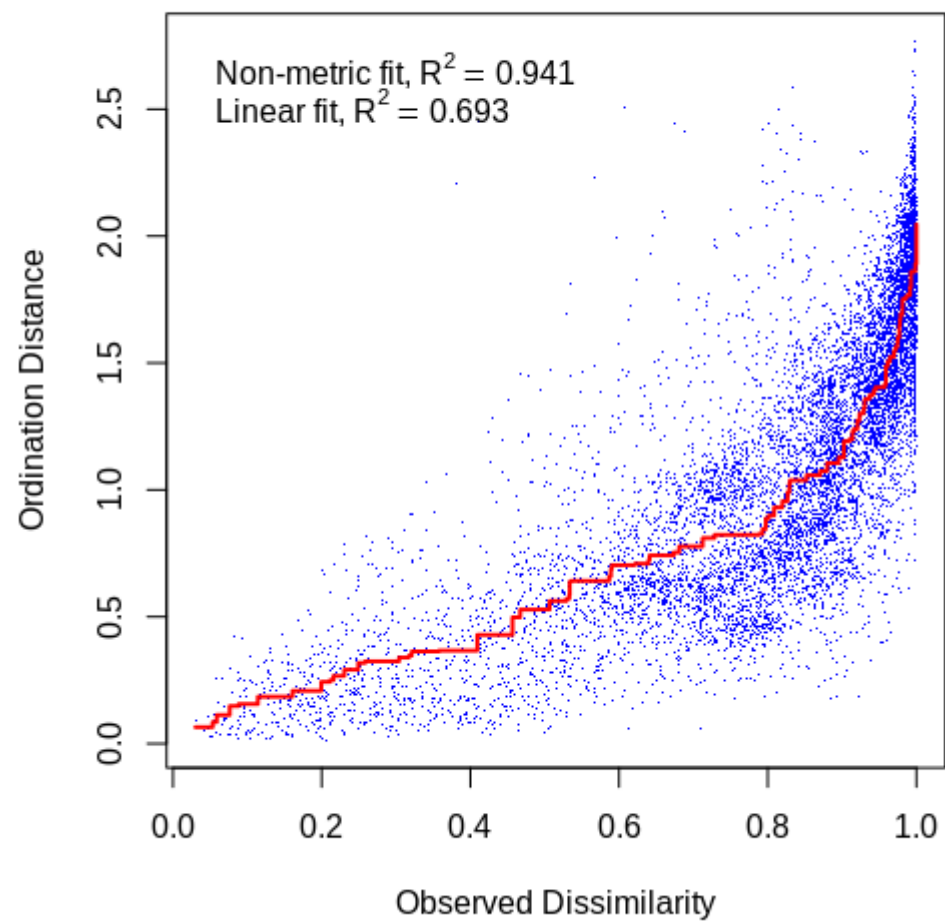

Supplement: Supplementary file 2 — Supplementary Information 2. [file 41598_2023_30764_MOESM2_ESM.pdf]
