# Supplementary material for: Determining the most accurate 16S rRNA hypervariable region for taxonomic identification from respiratory samples
Source: Sci Rep. 2023 Mar 9;13:3974. doi: 10.1038/s41598-023-30764-z (PMC9998635; doi:10.1038/s41598-023-30764-z)
Supplement: Supplementary file 3 — Supplementary Information 3. [file 41598_2023_30764_MOESM3_ESM.docx]

**Determining the most accurate 16S rRNA hypervariable region for taxonomic identification from respiratory samples**

Ruben López-Aladid* (1, 2, 3), Laia Fernández-Barat*^¶^ (1, 2, 3), Victoria Alcaraz-Serrano (1, 2, 3), Leticia Bueno-Freire (1, 2, 3), Nil Vázquez (1, 2, 3), Roque Pastor-Ibáñez (4), Andrea Palomeque (1, 2, 3), Patricia Oscanoa (1, 2, 3), Antoni Torres^¶^ (1, 2, 3).

1. Cellex Laboratory, CibeRes (Centro de Investigación Biomédica en Red de Enfermedades Respiratorias, 06/06/0028), Institut d'Investigacions Biomèdiques August Pi i Sunyer (IDIBAPS).
2. School of Medicine, University of Barcelona, Barcelona, Spain.
3. Department of Pneumology, Thorax Institute, Hospital Clinic of Barcelona, Spain.
4. Group of Genomics and Pharmacogenomics in HIV, Laboratory of Retrovirology and Viral Immunopathogenesis. Hospital Clinic of Barcelona.

^*^ Contributed equally

^¶^ Corresponding authors: Antoni Torres, MD, PhD ([atorres@clinic.cat](mailto:atorres@clinic.cat)) and Laia Fernández-Barat, PhD ([lfernan1@clinic.cat](mailto:lfernan1@clinic.cat))

**SUPPLEMENTAL MATERIALS**

**Supplementary Material S1. Amplicon Sequence variants of the Microbial community standard control according to the different hypervariable regions of the 16S gene.** Microbial community standard control from Zymobiomics have been grouped and averaged by each hypervariable region, and taxonomic composition is shown on the genus level. A total of 8 ASVs were obtained with 93.915 taxa counts in each hypervariable region**.**

**Supplementary Material S2.** Stress plot of NMDS2 ordination for beta diversity using Bray Curtis dissimilarity as a statistical measure used to quantify the compositional dissimilarity between two samples or groups. Related to evaluating ordination stress a screen plot showing the decrease in ordination stress with an increase in the number of ordination dimensions allowed. Finding the "breakpoint" can instruct selection of a minimum number of dimensions. If there is no breakpoint and adding more dimensions leads to a small, linear decrease in stress, the data set may not be suited to low-dimensional ordination. Shepard stress plot showing the relationship between the actual dissimilarities between objects (from the original dissimilarity matrix) and the ordination distances (i.e., the distances on the final plot). If these are well correlated, the ordination stress will be low and the visualization trustworthy.
